# Detecting Unilateral Phrenic Paralysis by Acoustic Respiratory Analysis

**DOI:** 10.1371/journal.pone.0093595

**Published:** 2014-04-09

**Authors:** José Antonio Fiz, Raimon Jané, Manuel Lozano, Rosa Gómez, Juan Ruiz

**Affiliations:** 1 Pneumology Service, Germans Trias i Pujol University Hospital, Badalona, Spain; 2 Institute for Bioengineering of Catalonia (IBEC), Barcelona, Spain; 3 Biomedical Research Networking Center in Bioengineering, Biomaterials, and Nanomedicine (CIBER-BBN), Barcelona, Spain; 4 Dept. ESAII, Universitat Politècnica de Catalunya (UPC), Barcelona, Spain; 5 Innovation Group, Health Sciences Research Institute of the Germans Trias I Pujol Foundation (IGTP), Badalona, Spain; Charité - Universitätsmedizin Berlin, Germany

## Abstract

The consequences of phrenic nerve paralysis vary from a considerable reduction in respiratory function to an apparently normal state. Acoustic analysis of lung sound intensity (LSI) could be an indirect non-invasive measurement of respiratory muscle function, comparing activity on the two sides of the thoracic cage. Lung sounds and airflow were recorded in ten males with unilateral phrenic paralysis and ten healthy subjects (5 men/5 women), during progressive increasing airflow maneuvers. Subjects were in sitting position and two acoustic sensors were placed on their back, on the left and right sides. LSI was determined from 1.2 to 2.4 L/s between 70 and 2000 Hz. LSI was significantly greater on the normal (19.3±4.0 dB) than the affected (5.7±3.5 dB) side in all patients (p = 0.0002), differences ranging from 9.9 to 21.3 dB (13.5±3.5 dB). In the healthy subjects, the LSI was similar on both left (15.1±6.3 dB) and right (17.4±5.7 dB) sides (p = 0.2730), differences ranging from 0.4 to 4.6 dB (2.3±1.6 dB). There was a positive linear relationship between the LSI and the airflow, with clear differences between the slope of patients (about 5 dB/L/s) and healthy subjects (about 10 dB/L/s). Furthermore, the LSI from the affected side of patients was close to the background noise level, at low airflows. As the airflow increases, the LSI from the affected side did also increase, but never reached the levels seen in healthy subjects. Moreover, the difference in LSI between healthy and paralyzed sides was higher in patients with lower FEV_1_ (%). The acoustic analysis of LSI is a relevant non-invasive technique to assess respiratory function. This method could reinforce the reliability of the diagnosis of unilateral phrenic paralysis, as well as the monitoring of these patients.

## Introduction

There are several causes of diaphragmatic dysfunction that can affect one or both muscles. The decrease in or cessation of motor activity can be caused by compression or section of the phrenic nerve in certain segments of the spinal cord [1]. The consequences of diaphragm dysfunction vary from the most serious cases of bilateral lesions that can require mechanical ventilation, to the mildest unilateral lesions that may to some extent impair breathing and in consequence exercise capacity [2], [3].

Diaphragm dysfunction due to phrenic paralysis has been studied with various techniques including x-ray, fluoroscopy, ultrasonography, and external or internal stimuli of the diaphragm. These techniques provide information regarding the position and mobility of the diaphragm muscle [4]–[7], but do not predict the degree of respiratory dysfunction [8].

On the contrary, breathing function can be measured by routine spirometry [9], [10]. Recently, Sokolowska et al. measured variations in breathing patterns in animals with bilateral phrenic paralysis, confirming that the measurement of breathing parameters could be an appropriate method to monitor this diaphragm dysfunction [11]. However, in cases of unilateral paralysis, spirometric function may be normal.

An alternative useful method to monitor breathing function is the measurement of pulmonary sounds [12]–[16]. In fact, it is known that airflow is correlated with lung sound intensity (LSI) [17], including in pulmonary conditions with restrictive ventilatory function [16].

Our hypothesis in the present study was that in patients with unilateral phrenic paralysis, the LSI on inspiration would be lower on the affected side than the healthy side. If this hypothesis were to be confirmed, measurements of LSI comparing the two sides could be useful to diagnose conditions associated with restricted thoracic mobility [6], as well as to monitor the response to specific physiotherapy treatments targeting the respiratory muscle.

## Materials and Methods

### Ethics Statement

The study was conducted in the Respiratory Function Laboratory at HUGTIP, since February 2011 to December 2013, and approved by the Human Research and Ethics Committee of the hospital. All participants gave written informed consent, following the World Medical Association's Declaration of Helsinki on Ethical Principles for Medical Research Involving Human Subjects.

### Study subjects

Patients with unilateral phrenic paralysis [18], who were previously diagnosed in the Department of Internal Medicine at Germans Trias i Pujol University Hospital (HUGTIP), were considered eligible for this study. All patients underwent chest radiography and computed tomography scanning of the chest, which reveal elevated hemidiaphragm on the affected side. Moreover, according to their medical history, most of the patients had previous thoracic or surgical trauma as the major cause of diaphragmatic paralysis. Only patient ID 2 had an unknown etiology. However, all patients related some level of functional dyspnea.

On the other hand, controls were selected from healthy subjects who had never been diagnosed of phrenic paralysis and had normal baseline spirometric values. According to these inclusion criteria, ten men with unilateral phrenic paralysis in a stable condition and ten controls (five men/five women) were included in the study for pulmonary function test and the acoustic respiratory analysis.

### Pulmonary function and lung sound testing

At baseline, lung function was measured by spirometry (Hyp'Air Compact, Medisoft). Table 1 shows baseline spirometric results from each subject. Measurements were obtained in accordance with established guidelines [19], and results compared to reference values [20].

**Table 1:** Subject Characteristics*.

Phrenic Nerve Paralysis Patients							
ID	Paralyzed Side	Age (yr)	Sex	BMI (kg/m^2^)	FVC L (% of predicted)	FEV_1_ L (% of predicted)	FEV_1_/FVC (%)
1	Right	46	Male	32.7	2.86 (56)	2.11 (54)	74.0
2	Left	52	Male	27.0	3.80 (70)	2.75 (66)	72.0
3	Left	38	Male	29.4	2.81 (55)	2.10 (51)	74.7
4	Left	70	Male	30.5	2.11 (56)	1.32 (49)	63.0
5	Left	41	Male	33.0	2.59 (57)	2.07 (57)	79.9
6	Left	74	Male	35.0	1.56 (40)	1.21 (42)	77.2
7	Left	54	Male	28.6	2.13 (50)	1.76 (53)	82.5
8	Right	70	Male	28.4	2.44 (53)	1.95 (57)	79.6
9	Left	59	Male	24.2	3.17 (69)	2.79 (80)	87.9
10	Left	69	Male	30.9	2.44 (64)	1.68 (62)	69.0
Mean		57		30.0	(57)	(57)	76.0
SD		13		3.2	(9)	(10)	7.1

Healthy Subjects							
ID	Paralyzed Side	Age (yr)	Sex	BMI (kg/m^2^)	FVC L (% of predicted)	FEV_1_ L (% of predicted)	FEV_1_/FVC (%)
1	None	28	Male	25.3	5.56 (97)	4.21 (90)	75.7
2	None	31	Male	22.3	4.50 (82)	3.85 (89)	86.0
3	None	48	Female	22.4	2.56 (76)	2.38 (92)	93.0
4	None	36	Male	24.4	5.38 (114)	4.05 (105)	75.3
5	None	24	Female	22.4	4.38 (92)	3.66 (94)	83.4
6	None	35	Female	23.0	4.45 (105)	3.63 (106)	81.5
7	None	31	Female	23.7	3.23 (91)	2.80 (95)	86.9
8	None	49	Female	26.5	3.25 (89)	2.66 (92)	82.0
9	None	22	Male	24.5	5.42 (91)	4.03 (82)	74.4
10	None	44	Male	24.4	5.43 (99)	3.84 (89)	70.8
Mean		35		23.9	(94)	(93)	80.9
SD		10		1.4	(11)	(7)	6.8

* BMI = body mass index; FVC = forced vital capacity; FEV_1_ = forced expiratory volume in 1 second. Anthropometric and spirometric values of ten patients with a unilateral phrenic paralysis and ten healthy subjects are shown. References for percentages of predicted normal values are from Roca et al. [20]. FEV_1_ and FVC in percentages were lower in all patients than in healthy subjects.

After this previous test, each subject was coached to progressively increase the airflow from shallow breathing to the deepest breaths they were able to, reaching 1.2 to 2.4 L/s [14]. Lastly, at the end of the respiratory test, subjects were asked to hold their breath for a few seconds in order to estimate background noise intensity (BNI). One recording of a total 120 seconds was obtained from each subject in a sitting position. Respiratory flow and sounds were acquired simultaneously during the test.

### Lung sounds and respiratory airflow measurements

Respiratory sounds were recorded using two contact microphones (TSD108, Biopac Systems, Inc.) with a frequency response of 35–3500 Hz. Microphones were positioned on the surface of the back, at each side of the spinal cord and 3 cm below the bottom tip of the shoulder blades. They were attached to the skin with double-sided adhesive discs, in a noninvasive way. In addition, respiratory airflow was recorded with a pneumotachograph (TSD107B, Biopac Systems, Inc.). Subjects wore a nose clip and breathed through the mouthpiece of the instrument.

Airflow and sound signals were amplified and filtered by hardware, before analog-to-digital conversion and acquisition. On the one hand, high- and low-pass filters with cut-off frequencies of 10 and 5000 Hz, respectively, were applied to respiratory sound signals, and they were amplified by a factor of 200. On the other hand, low-pass filter with a cut-off frequency of 10 Hz was applied to the airflow signal, and this was amplified by a factor of 1000. Then, both sound and flow signals were recorded at a sample rate of 12500 Hz using a 16-bit analog-to-digital converter (MP150, Biopac Systems, Inc.). Since this study is only focused on normal pulmonary sounds, whose bandwidth of interest is below 2000 Hz, respiratory sound signals were digitally filtered using a combination of 8th order Butterworth high- and low-pass filters with cut-off frequencies of 70 and 2000 Hz, respectively.

### Lung sound analysis

Respiratory sound signals were automatically segmented by extracting respiratory phases from the airflow signal. Respiratory cycles in which the flow reached at least 0.35 L/s were considered valid cycles. In order to avoid false detections caused by background noise, two thresholds of 0.2 and 4 seconds were established for minimum and maximum durations of breathing phases, respectively, according to time duration of normal respiratory cycles. In addition, a threshold of 0.5 seconds was fixed for the maximum time interval between the end of inspiration and the beginning of the corresponding expiration. All cycles not meeting these criteria were rejected. The final dataset for each subject was formed by audio-visual selection of pairs of sound signals, one from each side, from the same inspiratory cycles, avoiding artifacts such as those from swallowing or rubbing.

Each inspiratory sound cycle was firstly classified according to the maximum airflow reached. For that purpose, the airflow scale was divided into intervals of 0.2 L/s, from 1.2 L/s upwards. Furthermore, only inspiratory sound segments corresponding to the top airflow interval, whose duration is at least 20% of cycle length, of each inspiratory cycle were used for assessing the LSI.

The LSI was calculated as the mean power, in the frequency band from 70 to 2000 Hz, obtained from the power spectral density (PSD) of each inspiratory sound segment, according to the following expression:
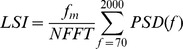
where *f_m_* is the sample rate, and *NFFT* is the number of points for the fast Fourier transform (FFT). Just as in some previous studies, which were focused on the intensity of respiratory sounds [14], [21], [22] the PSD was calculated using Welch's periodogram, with a Hanning window of 1000 data samples (80 ms), a 50% overlap between adjacent segments, and 1024 points for the FFT. The same method was applied to apnea segments from both left and right sides, in order to calculate the mean background noise intensity (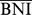). The resultant LSI values from all inspiratory sound segments were expressed in dB with respect to this 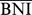. Having calculated the LSI, each subject was characterized by the relationship between the LSI and the airflow on both left and right sides. In addition, the LSI was averaged over the airflow range 1.2–2.4 L/s, in order to obtain a mean LSI for each side. Normality in the mean LSIs of both sides, from patients and healthy subjects, as well as in their differences was tested with a Lilliefors test. Since we did not know the parameters of the hypothesized distributions, and those parameters must be estimated from the data sample, the Lilliefors test was preferable. On the other hand, the statistical differences were tested between: 1) the mean LSIs of both sides, and 2) the differences in the mean LSIs of both sides from patients and healthy subjects. Since normality could not be assumed in all cases, and the sample size (n = 20) was small, a non-parametric test, such as the Wilcoxon rank sum test, was used to check for statistical differences.

## Results

### Lung sound intensity in unilateral phrenic paralysis

Acoustic and spirometric parameters were analyzed in patients and healthy subjects. As shown in Table 1, eight patients had left side paralysis (ID 2–7, 9, and 10) and two patients had right side paralysis (ID 1 and 8). Regardless of the side affected, all patients had lower FVC (57±9%) and FEV_1_ (57±10%) values than healthy subjects, in whom the percentages were 94±11% and 93±7%, respectively.

**Figure 1:**
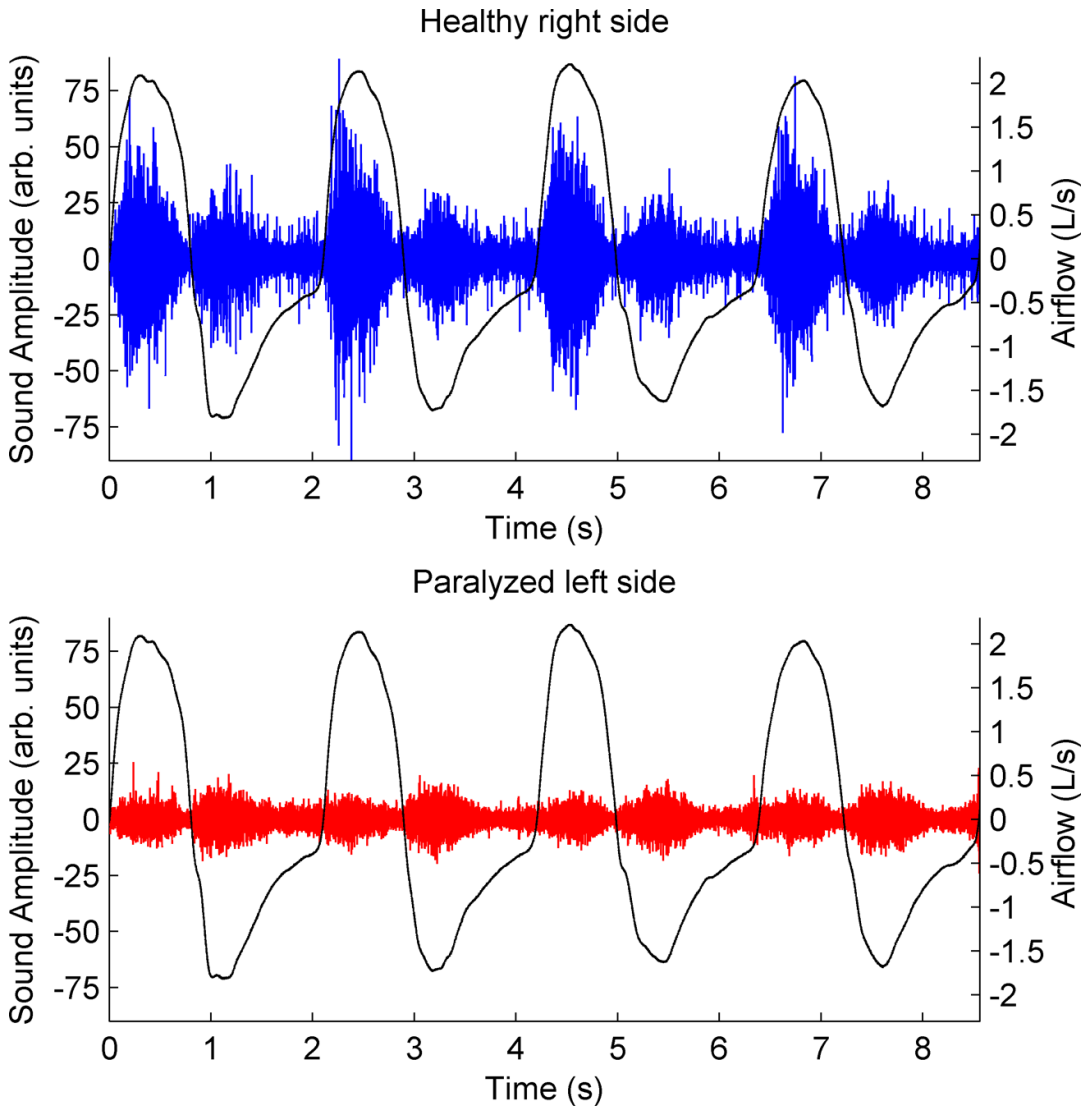
Airflow and lung sound signals. Airflow signal (black) and the corresponding lung sound signals, in arbitrary units, for both right (blue) and left (red) sides, in a patient with left side phrenic paralysis (ID 4). Sound amplitudes from the left side were lower than those from the healthy right side.

**Figure 2:**
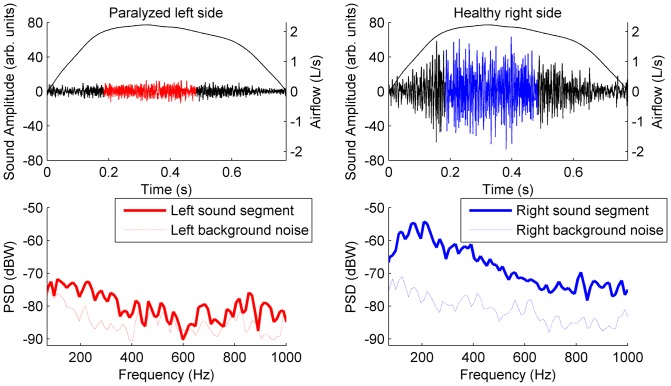
Power spectral density of lung sounds from a patient. Airflow (L/s), lung sound signals (arbitrary units), and the corresponding power spectral densities (dBW), for both sides of an inspiratory cycle from a patient with left side phrenic paralysis (ID 4). Solid and dotted lines in the PSDs correspond to the central sound segments and the background noise segments from both sides, respectively.

With regard to lung sounds, the signal amplitude was much lower on the paralyzed side than the healthy side, in patients with unilateral phrenic nerve paralysis, as shown in the example from Figure 1. It contains the lung sound and the airflow signals from a patient with left phrenic paralysis (ID 4). Accordingly, the magnitude of the PSDs from both sides, and the consequent signal powers, are quite different, as shown in Figure 2. It exhibits the PSDs from two inspiratory sound segments, one from each side, of an inspiratory cycle from patient ID 4. As shown, the PSD of the right sound segment (healthy side) is a long way from the PSD of the right background noise segment, in all the frequency range. On the other hand, the PSD of the left sound segment (affected side) is slightly above the left background noise. As a result, the LSI calculated from the PSD of the healthy side is much larger than the affected side.

The aforementioned pattern was confirmed by comparison of the acoustic parameters in all patients with unilateral phrenic paralysis (Table 2). Calculation of the 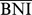 from the BNI for left and right sides allowed us to express the mean LSI from each side in dB with respect to the same reference value. In addition, the mean LSI was calculated for both sides from the same set of cycles.

**Table 2:** Acoustic Characteristics*.

Phrenic Nerve Paralysis Patients							
ID	BNI_Healthy_ (µW)	BNI_Paralyzed_ (µW)	BNI_Mean_ (µW)	LSI_Healthy_ (dB)*	LSI_Paralyzed_ (dB)*	LSI_Healthy_ - LSI_Paralyzed_ (dB)	# Left and Right Cycles
1	15.5	7.8	11.6	16.6	6.2	10.4	30
2	8.1	7.9	8.0	13.6	3.7	9.9	28
3	2.4	1.8	2.1	21.2	4.8	16.3	30
4	16.0	3.2	9.6	16.5	3.5	12.9	30
5	7.8	5.5	6.7	13.4	2.0	11.4	18
6	15.2	3.6	9.4	21.5	0.2	21.3	18
7	4.7	3.3	4.0	24.2	10.2	13.9	34
8	9.5	6.8	8.1	23.9	7.8	16.1	21
9	28.0	14.2	21.1	21.8	11.0	10.8	18
10	14.4	9.5	11.9	20.1	7.9	12.2	27
Mean	12.2	6.4	9.3	19.3	5.7	13.5	25
SD	7.3	3.7	5.2	4.0	3.5	3.5	6

Healthy Subjects							
ID	BNI_Left_ (µW)	BNI_Right_ (µW)	BNI_Mean_ (µW)	LSI_Left_ (dB)*	LSI_Right_ (dB)*	LSI_Right_ – LSI_Left_ (dB)	# Left and Right Cycles
1	1.2	4.7	2.9	12.8	15.8	3.0	40
2	9.0	3.5	6.2	8.4	8.9	0.5	44
3	20.2	26.7	23.4	23.8	24.9	1.1	16
4	0.8	1.9	1.4	13.3	17.9	4.5	30
5	1.7	3.9	2.8	13.5	17.1	3.6	28
6	1.5	25.7	13.6	11.0	13.2	2.2	42
7	6.6	5.2	5.9	26.6	27.0	0.4	26
8	1.1	2.2	1.6	20.5	22.1	1.6	47
9	1.8	4.7	3.3	10.9	12.5	1.6	14
10	1.5	3.0	2.2	10.3	15.0	4.6	22
Mean	4.5	8.2	6.3	15.1	17.4	2.3	31
SD	6.2	9.6	7.0	6.3	5.7	1.6	12

* With respect to mean background noise intensity (BNI_Mean_); BNI = background noise intensity; LSI: lung sound intensity; #: number of cycles analyzed. Acoustic characteristics are shown for ten patients with a unilateral phrenic paralysis and ten healthy subjects. Absolute differences in mean inspiratory LSI (|LSI_Left_-LSI_Right_|) were higher in patients than in healthy subjects.

In healthy subjects, the mean LSI was much higher than the 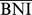 on both left (15.1±6.2 dB) and right (17.4±5.7 dB) sides. However, patients had mean LSIs only a few dBs above the 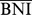 on the affected side (5.7±3.5 dB) while their mean LSIs on the healthy side (19.3±4.0 dB) were not significantly different from the values measured in the healthy participants. To show this trend clearly, we calculated the difference between the mean LSI of each side.

**Figure 3:**
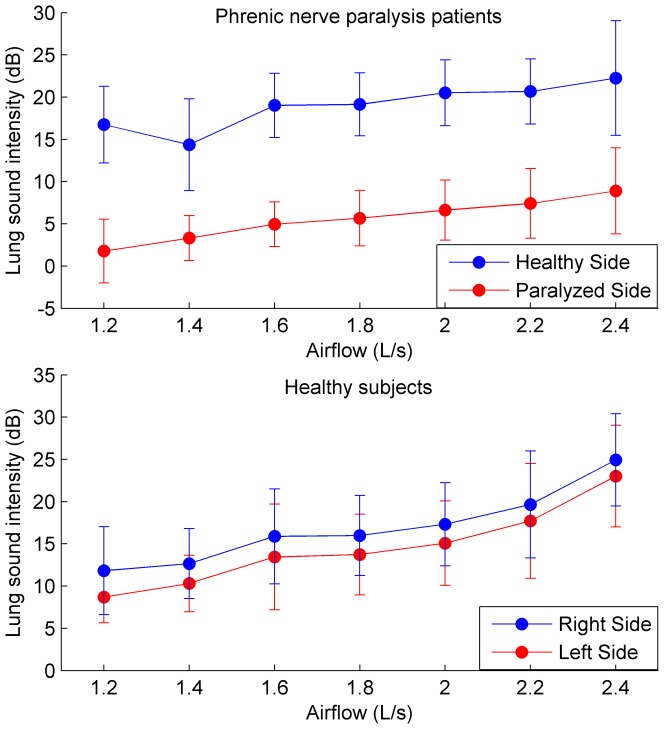
Comparison of inspiratory LSI-Airflow relationship between both hemithoraxes in patients and healthy subjects. Mean inspiratory LSI (dB) as a function of airflow (L/s), from the ten patients and the ten healthy subjects. All values are the mean ± SD.

Figure 3 shows the mean LSI, for each side, as a function of airflow level in all patients, and all healthy subjects. On the one hand, considerable differences, of more than 13 dB, can be seen between the LSI from the affected and healthy sides. On the other hand, differences in LSI between the sides are less than 3 dBs in healthy subjects. It should be noted that the LSI from the affected sides are close to the 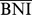 (0 dB) at low airflows. As the airflow increases, the LSI from the affected sides does also increase, but never reaches the levels seen in healthy subjects.

Furthermore, Figure 3 shows a clear linear relationship between the LSI and the airflow level. This sound-flow relationship has been reported in some previous studies [23]–[25], and it usually follows a power law. In a logarithmic scale (dB), this relationship can be formulated by a linear equation:
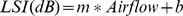
where m is the slope of the line, and b is the y-intercept. As shown in Table 3, all LSI-airflow relationships from Figure 3 can be properly expressed by a linear equation. Moreover, there is a clear difference between the slope of healthy subjects (around 5 dB/L/s) and patients (around 10 dB/L/s), independently of the analyzed side.

**Table 3:** Linear regression parameters*.

	Healthy subjects		Patients	
	Right side	Left side	Paralyzed side	Healthy side
R^2^	0.91	0.93	0.98	0.79
Slope (dB/L/s)	9.78	10.61	5.58	5.48

* Corresponding to graphs from Figure 3.

**Figure 4:**
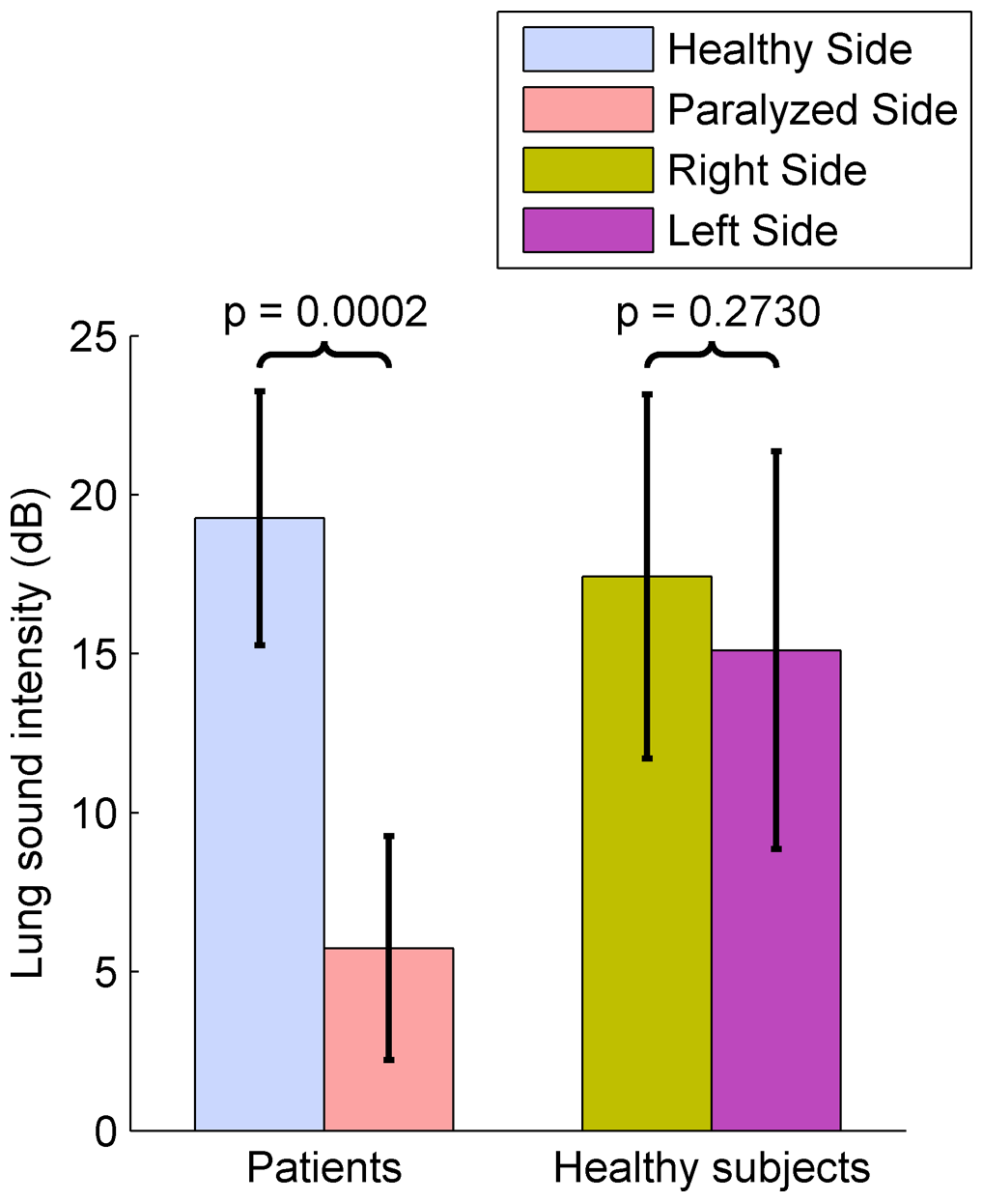
Comparison of mean inspiratory LSI between both hemithoraxes in patients and healthy subjects. Mean inspiratory LSI (dB) in healthy and paralyzed sides (ten patients), and in right and left sides (ten healthy subjects). The mean LSI from patients is significantly higher in healthy side than paralyzed side (p = 0.0002). On the contrary, there are not significant differences between mean LSI from both hemithoraxes in healthy subjects (p = 0.2730).

The mean inspiratory LSI from both sides of patients and healthy subjects has been statistically analyzed, as shown in Figure 4. The null hypothesis that the mean LSIs were normally distributed was accepted as much for both healthy and paralyzed sides in patients (p = 0.4135 and 0.9436, respectively), as for the right side in healthy subjects (p = 0.5790). However, the null hypothesis was rejected for the left side in healthy subjects (p = 0.0104).

The Wilcoxon rank sum test showed that the mean LSIs of healthy and paralyzed sides in all patients were statistically different (p = 0.0002). On the contrary, the difference between the mean LSIs of right and left sides in all healthy subjects was not statistically significant (p = 0.2730).

### Lung sound intensity differences and FEV_1_ relationship

**Figure 5:**
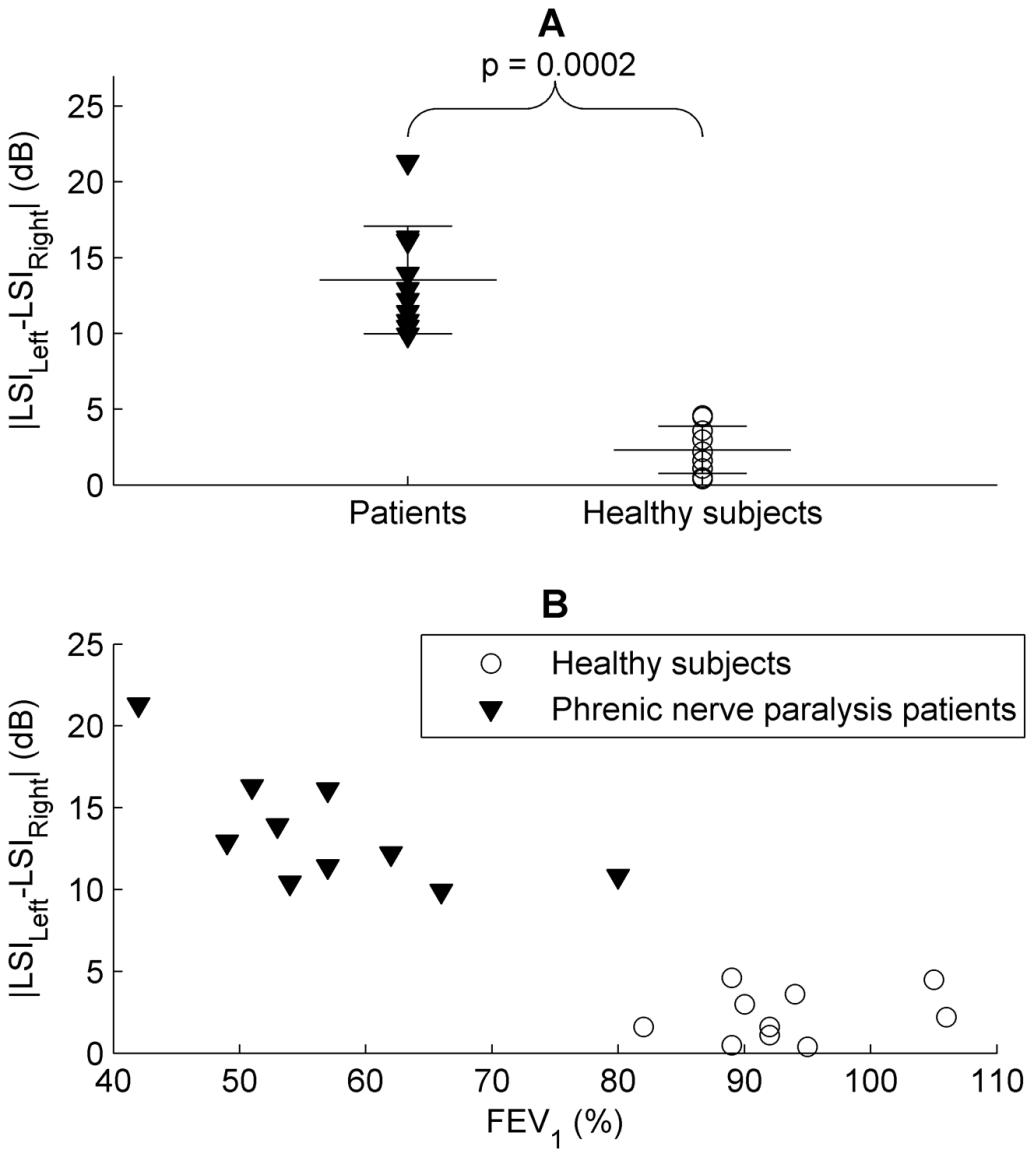
LSI differences and FEV_1_ relationship in patients and healthy subjects. A: Mean inspiratory LSI difference (dB) between both hemithoraxes, in ten patients with phrenic nerve paralysis and ten healthy subjects. The LSI difference was higher in patients than healthy subjects (p = 0.0002). Solid lines indicate the mean and SD for each group. B: Mean inspiratory LSI difference as a function of FEV_1_ in patients and healthy subjects.

Figure 5-A shows the absolute value of the differences between the mean LSIs of both sides (|LSI_Left_-LSI_right_|). In this case, the null hypothesis that the differences were normally distributed was accepted as much in patients (p = 0.6078), as in healthy subjects (p = 0.4693).

The Wilcoxon rank sum test showed that the differences between the mean LSIs of both sides were statistically significant in both groups (p = 0.0002). Moreover, it was found that there was a clear cut off around 6–8 dB which distinguished patients from healthy subjects.

Figure 5-B illustrates the relationship between the mean LSI difference and FEV_1_, showing high differences in the LSI and low FEV_1_ in patients with phrenic nerve paralysis. Moreover, in patients there is an inverse relationship between the two parameters, namely the lower the FEV_1_, the higher the mean LSI difference. In contrast, healthy subjects have low mean LSI differences, and there is no any clear relationship between these LSI differences and the corresponding FEV_1_.

## Discussion and Conclusions

Our study shows that patients with unilateral phrenic nerve paralysis have a lower inspiratory sound intensity on the affected side than the healthy one. We did not analyze expiratory sounds due to the lower values of expiratory intensity with respect to inspiratory values at isoflows [14]. This study illustrates the potential of lung acoustic analysis for the diagnosis and management of these patients.

Respiratory sounds are an alternative method to measure both pulmonary [16] and diaphragmatic function. Some previous studies reported decreased breath sound on the affected side in patients with unilateral phrenic paralysis [18], [26], [27], but they were assessed by traditional auscultation. However, there are no references about quantitative analysis of respiratory sounds for the diagnosis of these patients. In what is related to laterality of respiratory sounds, they have been used to distinguish between bilateral and unilateral lung ventilation in intubated patients [28]. Nevertheless, many studies have analyzed the differences between the LSI of both sides in healthy subjects [14], [29], [30], [31], thus reporting slight differences of a few dB. In any case, sound analysis can detect differences in airflow entering the two sides of the thoracic cage in diseases that affect respiratory ventilation, and our study demonstrates this for the case of unilateral phrenic nerve paralysis. Consistently, we found a clear cut-off in the mean differences of LSI between the two sides in healthy subjects and patients.

In addition to unilateral phrenic paralysis, it has been recognized by other authors that lung sound analysis is also a very useful technique to study many others pulmonary diseases [21], [22], [32], [33].

When the diaphragm is paralyzed, it does not have an influence on expansion of the homolateral lung and breathing is maintained by accessory muscles such as those of the chest wall. The movement of the paralyzed hemidiaphragm is determined by the balance between the change in pleural pressure and the shortening of the healthy hemidiaphragm. This is manifested by a cranial displacement of the ipsilateral hemidiaphragm and a small caudal displacement of the contralateral hemidiaphragm [6]. Such a retraction is ineffective for respiration and has been related to patient dyspnea [34].

It has been suggested that the airflow to dependent areas of the lung is directed by the diaphragm and non-dependent areas by the intercostal muscles [35]. The gas flow to the dependent areas of the paralytic side would therefore be lower than that to the healthy side.

In this study, there was considerably less airflow entering the dependent areas of the pathological side, measured in an indirect way by the quantification of the LSI. Specifically, the LSI of the affected side was close to the level of the background noise for a low airflow rate, as seen in Figure 3, while the signal from the healthy side remained a long way from the background noise at all measured flow rates. Although the BNIs from both sides are slightly different, it is not relevant for the results of this study, since the 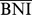 is used as the unique reference value in order to express the LSI in dB.

In addition, pulmonary perfusion is redistributed from the base toward the apex in these patients [36]. The result of this pathological situation is that the work of breathing (measured in terms of oxygen uptake) is increased, which suggests that intercostal muscle breathing is less efficient than diaphragm breathing [37]. Spirometric changes have been widely commented on in the literature. In our study, spirometry values of patients with phrenic paralysis were low with respect to normal reference values, as has been found previously in other studies [9], [38].

With respect to traditional techniques to diagnose the unilateral phrenic paralysis, they include: x-ray imaging, fluoroscopy, ultrasonography, and phrenic nerve stimulation [18], [39]. Usually, unilateral phrenic paralysis is diagnosed by a combination of these techniques, since none of them is totally concluding by itself. Of these techniques, x-ray imaging is the simplest and it has some obvious limitations: it uses ionizing radiation, and it does not allow us to assess the diaphragm or the pulmonary function of patients. Moreover, in unilateral diaphragmatic paralysis, the sensitivity of plain chest radiograph is as high as 90%, whereas its specificity is, however, low (44%) [39].

Fluoroscopy and the external or internal stimuli of the diaphragm allow evaluating the diaphragm mobility [4]. However, fluoroscopy also makes use of x-rays to obtain dynamic images of the diaphragm, and both fluoroscopy and stimuli of the diaphragm are invasive techniques. Moreover, none of these methods provides information about the pulmonary function.

Ultrasonography is an alternative non-invasive technique to assess the diaphragmatic function [5], [40], since it works on ultrasounds. Nevertheless, just as the aforementioned techniques, ultrasounds do not provide any data about the pulmonary function of patients. Moreover, ultrasonography is operator dependent and requires significant expertise [39].

Recently, a new non-invasive method has been proposed to measure the movements of the thoracic wall [41]. This new method makes use of a motion analysis system, which is called optoelectronic plethysmography. It was used to estimate the total rib cage volume, as well as its changes in both healthy and paralyzed sides.

In this study, the potential of acoustic respiratory analysis for detecting unilateral phrenic paralysis has been clearly shown. Despite a relatively small population has been analyzed, the results from 20 subjects (10 patients and 10 healthy subjects) reinforce the reliability of the proposed method. On the other hand, in the database, there is a slight difference in the male-female ratio between patients and healthy subjects, but gender is not a relevant factor in the analysis of normal lung sound intensity [42]. However, further studies will be needed to clinically validate this technique as a new complementary tool for phrenic paralysis diagnosis.

In conclusion, measurement of LSI can provide quantitative information about the extent of impairment of respiratory function in patients with unilateral phrenic nerve paralysis. In these patients, LSI is an indirect measure of the airflow that enters the lungs, this being lower on the affected side due to inefficient diaphragmatic muscle function. This technique represent a step forward in the diagnostic procedure of unilateral phrenic nerve paralysis, since it has some advantages with respect to current techniques: non-invasiveness, objectivity, simplicity, easiness and cost. The acoustic respiratory analysis, in conjunction with spirometry, could reinforce the reliability of the diagnosis of unilateral phrenic paralysis.

Regarding the future use of the method, its major application is the non-invasive assessment of respiratory function, providing objective information of the affected side. Therefore, the method offers the capability for long-term monitoring of recovery in respiratory function in patients who undergo physical therapy [43]. These patients are regularly monitored in order to check whether the physical therapy is improving their pulmonary function in the affected side or not. In this context, the advantages of the proposed technique gain relevance since several and repeated tests are required for the long-term monitoring of these patients.
